# Drosophila ezoana uses an hour‐glass or highly damped circadian clock for measuring night length and inducing diapause

**DOI:** 10.1111/phen.12165

**Published:** 2016-09-08

**Authors:** Koustubh M. Vaze, Charlotte Helfrich‐Förster

**Affiliations:** ^1^ Neurobiology and Genetics, Theodor‐Boveri Institute, BiocenterUniversity of WürzburgWürzburgGermany

**Keywords:** Circadian clock, damped‐oscillator‐model of photoperiodic clock, diapause, Drosophila, hour‐glass, Nanda–Hamner, photoperiodism

## Abstract

Insects inhabiting the temperate zones measure seasonal changes in day or night length to enter the overwintering diapause. Diapause induction occurs after the duration of the night exceeds a critical night length (CNL). Our understanding of the time measurement mechanisms is continuously evolving subsequent to Bünning's proposal that circadian systems play the clock role in photoperiodic time measurement (Bünning, 1936). Initially, the photoperiodic clocks were considered to be either based on circadian oscillators or on simple hour‐glasses, depending on ‘positive’ or ‘negative’ responses in Nanda–Hamner and Bünsow experiments (Nanda & Hammer, 1958; Bünsow, 1960). However, there are also species whose responses can be regarded as neither ‘positive’, nor as ‘negative’, such as the Northern Drosophila species Drosophila ezoana, which is investigated in the present study. In addition, modelling efforts show that the ‘positive’ and ‘negative’ Nanda–Hamner responses can also be provoked by circadian oscillators that are damped to different degrees: animals with highly sustained circadian clocks will respond ‘positive’ and those with heavily damped circadian clocks will respond ‘negative’. In the present study, an experimental assay is proposed that characterizes the photoperiodic oscillators by determining the effects of non‐24‐h light/dark cycles (T‐cycles) on critical night length. It is predicted that there is (i) a change in the critical night length as a function of T‐cycle period in sustained‐oscillator‐based clocks and (ii) a fixed night‐length measurement (i.e. no change in critical night length) in damped‐oscillator‐based clocks. Drosophila ezoana flies show a critical night length of approximately 7 h irrespective of T‐cycle period, suggesting a damped‐oscillator‐based photoperiodic clock. The conclusion is strengthened by activity recordings revealing that the activity rhythm of D. ezoana flies also dampens in constant darkness.

## Introduction

Photoperiodic time measurement is referred to as the ability to measure seasonal variation in day/night length and is an integral feature of the seasonal adaptations in organisms inhabiting temperate zones. Seasonal adaptations include physiological modulations that typically culminate in the regulation of growth and reproduction. Although summer conditions typically induce growth and reproduction, winter conditions arrest the latter. Such responses are respectively known as ‘long’ and ‘short’ day responses (Bradshaw & Holzapfel, 2007; Nelson *et al*., 2009).

Photoperiodic time measurement has been studied for almost a century; however, the mechanism underlying time measurement (i.e. the clock) remains elusive. In 1936, Erwin Bünning proposed a role for the circadian clock in photoperiodic time measurement. Bünning's model assumes two distinct phases of the circadian cycle, namely the *photophil* and *scotophil* phases, whereby the exposure of the *scotophil* phase to light is dependent on the photoperiod. He proposed that flowering is induced in the common bean *Phaseolus vulgaris* only when its *scotophil* phase is not exposed to light (Bünning, 1936). A modified version of this principle of photoperiodic time measurement was later proposed by Pittendrigh & Minis (1964) as the ‘external coincidence model’. Bünning's idea remained neglected for a long time until two experimental protocols, namely the Nanda–Hamner and Bünsow experiments (Nanda & Hamner, 1958; Bünsow, 1960), implicated circadian involvement in photoperiodic time measurement (Vaz Nunes & Saunders, 1999; Saunders, 2010; Saunders & Bertossa, 2011). In Nanda–Hamner experiments, study organisms are subjected to light/dark (LD) cycles of periods typically ranging from 16 to 84 h with a fixed light phase of 8–12 h and variable hours in the remaining dark phase to make up the required period length (Vaz Nunes & Saunders, 1999). Bünsow experiments employ LD cycles of 48 or 72 h period consisting of 12 h of light and of a variably long night. The long night is systematically interrupted, in different experimental subsets, by a 1‐ or 2‐h long scanning light pulse (Vaz Nunes & Saunders, 1999). Some species exhibit rhythmic variation in their short‐day response as a function of (i) the period of LD cycle in Nanda–Hamner experiments and (ii) the time of the scanning light pulse in Bünsow experiments. After the first application of these experimental protocols, a rhythmic response (=positive response) was instantly recognized as evidence for recurring ‘scotophil phases’ as envisioned by Bünning, especially because these short‐day effects peak at LD periodicities of 24, 48 and 72 h in Nanda–Hamner experiments and in light regimes with scanning pulses separated by 24 h in Bünsow experiments. However, in many other species, the short‐day effect reaches its maximum beyond a particular LD periodicity in Nanda–Hamner experiments or a scanning pulse in Bünsow experiments and remains stable thereafter (=negative response). Such responses are typically taken as evidence for a simple non‐oscillatory night length measuring hour‐glass (Vaz Nunes & Saunders, 1999). However, the binary classification of photoperiodic clocks (circadian versus hour‐glass) became obsolete after Lewis & Saunders (1987) proposed the ‘damped circadian oscillator model’. Saunders & Lewis (1987) were able to simulate positive and negative Nanda–Hamner and Bünsow phenotypes by simply adjusting the degree of oscillator dampening in the model, suggesting that the dampening tendency of the oscillator is a primary cause of the positive and negative responses.

**Figure 1:**
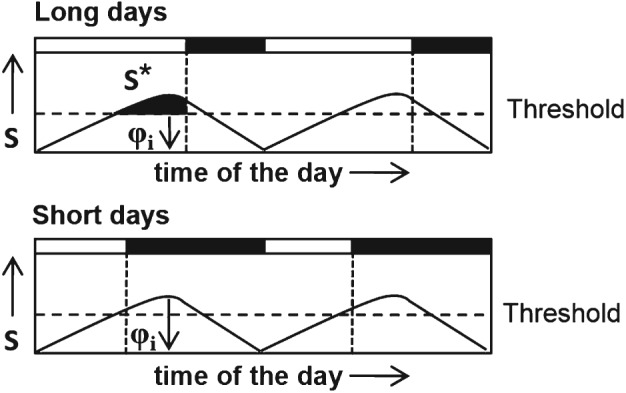
The external coincidence model (Pittendrigh & Minis, 1964). The external coincidence model assumes a circadian rhythm in the abundance of a hypothetical substrate (S), which is converted into its active form S* by a light‐dependent enzymatic reaction. The S level (and hence S*) crosses a threshold and reaches its maximum at a certain time of the day, which is termed as the photo‐inducible phase (ϕ_i_). The coincidence of ϕ_i_ with light under long days generates a sufficient amount of S* leading to an induction of long‐day response such as reproduction. In other words, the photo‐inducible phase (ϕ_i_) lies in the light phase. Under short days, ϕ_i_ lies in darkness, which leads to short‐day responses such as diapause.

**Figure 2:**
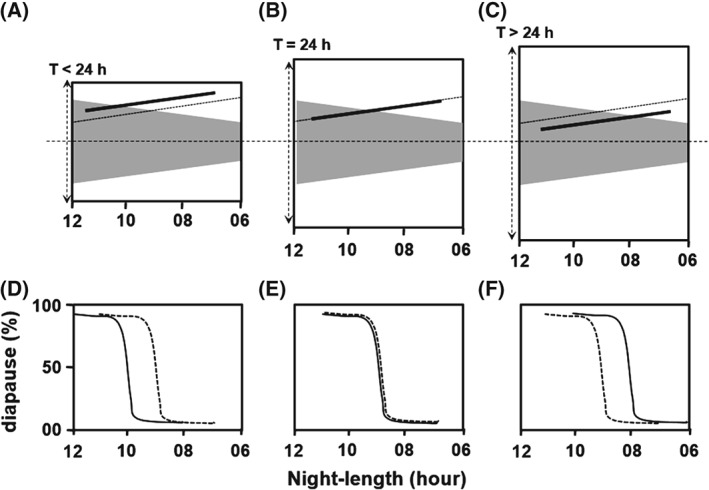
Expected change in the photo‐inducible phase (ϕ_i_) with photoperiod and Zeitgeber period (T). (A)–(C) Qualitative trends in the phase of ϕ_i_ as a function of night length at three Zeitgeber periods T < 24 h, T = 24 h and T > 24 h. (D)–(F) Corresponding change in the critical night lengths (CNL). For each Zeitgeber periodicity, the night is depicted as a grey area, with night length becoming shorter from left to right (starting with 12 h and ending with 6 h). The phase of ϕ_i_ and the photoperiodic response curves of the sustained oscillator based clock are depicted by a thick black line, whereas those of the damped oscillator based clock are indicated by a dashed line. For example, at T = 24 h (B), the transition of ϕ_i_ (thick black line) from dark to light occurs at a night length of approximately 9 h and thus has a CNL of approximately 9 h (E). On the other hand, the delayed occurrence of ϕ_i_ at T < 24h (thick line is pushed up) would make the CNL longer (e.g. 10 h) (A, D), whereas the earlier occurrence of ϕ_i_ (thick line is pushed down) would result in a shorter CNL at T > 24h (e.g. 8 h) (C, F). The ϕ_i_ of the damped oscillator based clock (dashed line) will not show such a dependency on T, thus leading to a constant CNL of 9 h at all the Ts. In conclusion, the photoperiodic response curves under T‐cycles can indicate whether the photoperiodic clock behaves as sustained circadian oscillator or a damped circadian oscillator.

Although, in theory, the damped and sustained oscillators closely simulate negative and positive responses, respectively (Saunders & Lewis, 1987), the proposed role of oscillator dampening in *positive* and *negative* Nanda–Hamner/Bünsow responses remains to be demonstrated empirically and the damped circadian oscillator model still remains as an open hypothesis. The absence of an empirical proof for the damped circadian oscillator model is primarily attributable to the lack of a means of identifying and/or manipulating the dampening tendency of the photoperiodic oscillator. In the present study, an experimental set‐up is proposed to identify the dampening tendency of the photoperiodic oscillator (damped versus self‐sustained), which may facilitate the experimental validation of the damped circadian oscillator model. The damped circadian oscillator model (Saunders & Lewis, 1987) formally adopts the ‘external coincidence model’ of Pittendrigh & Minis (1964) as the basis of photoperiodic time measurement (Fig. 1). The external coincidence model assumes a circadian rhythm of a photo‐inducible phase (ϕ_i_) (Fig. 1). The coincidence of ϕ_i_ with light under long days induces long‐day effects (reproduction), whereas its protection from light under short days leads to short‐day effects (diapause) (Fig. 1). The circadian regulation implies that the phase of ϕ_i_ would change with the period (T) of the Zeitgeber cycle. Pittendrigh & Minis (1964) therefore report the application of Zeitgeber cycles of different periods to test the external coincidence model, known as T‐cycle experiments. In the present study, a modification of the T‐cycle experiments is proposed, in which we predict distinct photoperiodic responses of the clock based on heavily damped and self‐sustained oscillators, respectively. The photoperiodic response curve (PPRC) typically describes the relationship between the incidence of short‐day responses and the photoperiod (or night length); here, the night length corresponding to the transition from short‐ to long‐day response is termed the ‘critical night length’. Figure 2 illustrates the conjectured correspondence between the transition of ϕ_i_ from dark to light and the critical night length. As noted above, the phase relationship ϕ_i_ is a function of *T* (Pittendrigh & Minis, 1964): ϕ_i_ is expected to be delayed in the case of *T* < 24 h and advanced when *T* > 24 h (Fig. 2). Consequently, we propose that, if the PPRCs are conducted under T‐cycles, a self‐sustained oscillator‐based photoperiodic clock would result in a predictable change in the critical night length as shown in Fig. 2. On the other hand, if the photoperiodic clock is based on a heavily damped oscillator, the oscillation is completely damped by the end of every cycle and will be restarted by the onset of darkness, causing ϕ_i_ to occur at a fixed number of hours afterwards (Pittendrigh, 1966). The critical night length should therefore not change with *T* in the case of a heavily damped oscillator. In the case of a slightly damped oscillator, critical night length is expected to change slightly with *T*. The modified T‐experiments may therefore provide a method of distinguishing between self‐sustained, slightly damped and heavily damped oscillator‐based photoperiodic clocks.

In the present study, the photoperiodic response in diapausing species of the *virilis* group *Drosophila* species *Drosophila ezoana* is investigated via the Nanda–Hamner protocol and modified T‐experiments. Furthermore, the dampening properties of the circadian oscillator are validated by recording the locomotor activity rhythms of individual flies under constant conditions. The results are discussed in the context of findings obtained in other insects.

## Materials and methods

### Fly material

All of the experiments were conducted with the *D. ezoana* strain 1240 J8 (isofemale line) established from the progeny of flies collected from Oulanka, Finland (66°N, 2°E) in the summer of 2008 (Salminen *et al*., 2015). The flies have been reared at laboratory room temperature (approximately 20 °C) under constant light (approximately 150 μW cm^−2^) on standard cornmeal–yeast–agar food. Eggs were collected over 2–3 consecutive days from parent vials containing five females and five males per vial. Freshly emerged adults were collected every day and maintained at a density of approximately 25–30 flies per food vial under rearing conditions. Approximately 4‐day‐old female flies were used to set all the experiments.

### Diapause assay

*Drosphila ezoana* exhibits photoperiodic adult reproductive diapause (Watabe, 1983) characterized by arrest of reproduction after exposure to appropriate environmental cues such as short‐day length (=long nights) and low temperature. Reproductive arrest manifests itself as the interruption of ovarian development, which leads to very small ovary size and suspension of egg development. Ovarian phenotype thus serves as reliable marker of diapause state.

To assay diapause incidence, four vials each containing 12 females and eight males (aged 4 days) were exposed to different LD regimes (Nanda–Hamner protocol or T‐cycles, see below) for 21 days at 17 °C with a food change once per week. At the end of the experiment, the flies were frozen at −20 °C and the females were dissected under a light microscope to examine the ovary phenotype. Females with a small ovary size without developing or mature eggs were counted as diapausing flies, whereas females with large ovaries with developing and mature eggs were marked as reproducing flies (Lankinen, 1986). Diapause incidence in the different LD regimes was expressed as a percentage of diapausing female flies from the four diapause vials.

All diapause assays were conducted in light‐proof cardboard boxes (16 × 26 × 15 cm^3^) fitted with ‘white’ light‐emitting diodes (for spectrum, see Schlichting *et al*., 2014). Light intensity inside the boxes was adjusted to approximately 145 μW cm^−2^ and the boxes were kept in a climate chamber maintained at a constant temperature of 17 °C.

### Nanda–Hamner experiments

The diapause incidence was studied in Zeitgeber cycles with periods (T) ranging from 18 to 84 h. Each cycle consisted of fixed 10 h of light and a variable number of hours in darkness to achieve the required T. The experiment was repeated three times and the diapause incidence was plotted as a function of T.

### Experimental design of the novel T‐cycle experiments

The diapause incidence was studied in a series of LD cycles with decreasing night lengths to construct the PPRCs, in which the percentage of diapause incidence was plotted as a function of night length (Fig. 2). Such PPRCs were conducted in LD cycles with a period (T) in the range 18–30 h (T18 to T30, respectively). Thus, the LD cycles were simultaneously varied for two aspects, namely night length and period (T).

Typically, diapause incidence is almost 100% under long nights and drops to almost 0% under short nights. The transition from diapause to the reproductive state is rather abrupt (Fig. 2). The night length corresponding to 50% diapause during this transition is taken as the value of critical night length. The critical night length was estimated by nonlinear regression analysis for each of the T‐cycles using the ‘drc’ package in r (Ritz & Streibig, 2005; R Development Core Team, 2008).

Two separate experiments were conducted in which the night length was varied within each T‐cycle, in two different ways. In Experiment 1, six LD cycle schedules were created within each of the T‐cycles, where night length was decreased in steps of 5% of the T‐cycle period between 75% and 50% in T18 and T21, 80–55% in T24, and 85–60% in T27 and T30 (Table 1). The diapause incidence was then plotted as a function of night length, expressed as a percentage of T, and absolute night length (h) to estimate the CNL for all the T‐cycles.

**Table 1:** Duration of light and dark phases under the different photoperiods and T‐cycles in Experiment 1.

Photoperiod (% of T‐cycle)	Light phase (h)					Dark phase (h)				
	T18	T21	T24	T27	T30	T18	T21	T24	T27	T30
85				22.95	25.5				4.05	4.5
80			19.2	21.6	24.0			4.8	5.4	6.0
75	13.5	15.75	18.0	20.25	22.5	4.5	5.25	6.0	6.75	7.5
70	12.6	14.7	16.8	18.9	21.0	5.4	6.3	7.2	8.1	9.0
65	11.7	13.65	15.6	17.55	19.5	6.3	7.35	8.4	9.45	10.5
60	10.8	12.6	14.4	16.2	18.0	7.2	8.4	9.6	10.8	12.0
55	9.9	11.55	13.2			8.1	9.45	10.8		
50	9.0	10.5				9.0	10.5			

In Experiment 2, the night length was varied from 5 to 10 h within each T‐cycle (Table 2). Diapause incidence was plotted against absolute night length and CNL was estimated for all the T‐cycles. The experiment was repeated three times to test whether the mean CNL differed across T‐cycles using two‐way analysis of variance (anova) in r (R Development Core Team, 2008).

**Table 2:** Duration of light and dark phases (h) under the three tested T‐cycles in Experiment 2.

Light regime	T18		T24		T30	
	Light phase	Dark phase	Light phase	Dark phase	Light phase	Dark phase
LD1	13	5	19	5	25	5
LD2	12	6	18	6	24	6
LD3	11	7	17	7	23	7
LD4	10	8	16	8	22	8
LD5	9	9	15	9	21	9
LD6	8	10	14	10	20	10

### Locomotor activity recordings

For activity recording, unmated female flies were collected on the first day after emergence by separating females from male flies under gaseous CO_2_ anaesthesia. Individual female flies were transferred into glass tubes (length 5 cm, diameter 7 mm) containing sugar‐agar medium (4% sucrose, 2% agar in water). They were kept for few days in constant light (LL) (150 μW cm^−2^) and then recorded in the *Drosophila* activity monitoring system (Trikinetics Inc., Waltham, Massachusetts) in constant darkness (DD).

### Analysis of activity data

To reveal the activity pattern of the flies, activity data were plotted as individual and average actograms using the imagej plug‐in actogramj (Schmid *et al*., 2011). To check for rhythmicity in the circadian range (16–30 h), the activity data of each individual fly was subjected to chi‐squared periodogram analysis. Periodogram analysis was carried out using continuous 10 days of data with a step size (data collection bin size) of 10 min. Furthermore, the number of days that each fly was rhythmic after the transfer from LL to DD was judged by eye for each single actogram.

## Results

### The Nanda–Hamner response of D. ezoana flies is neither positive, nor negative

**Figure 3:**
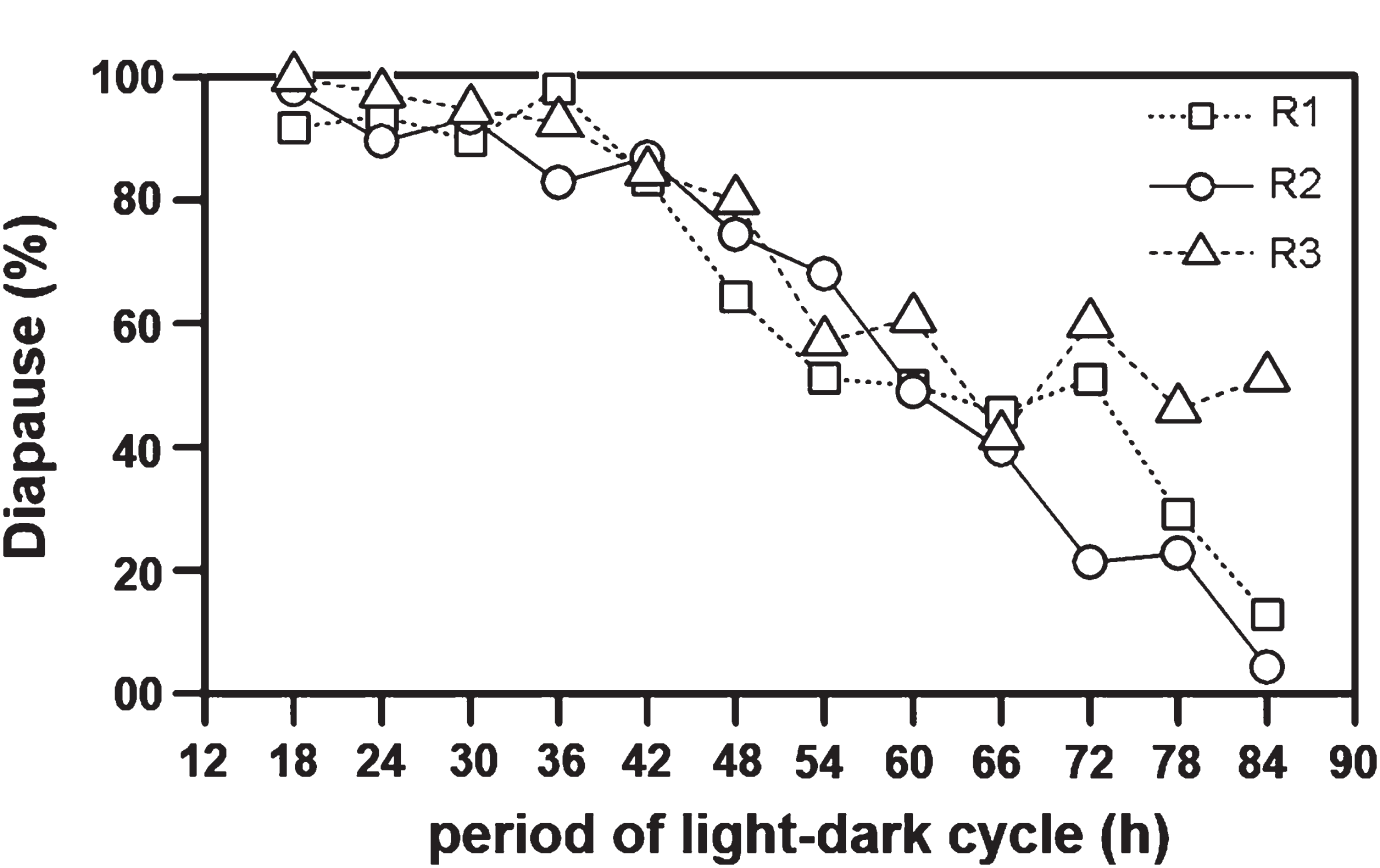
Diapause incidence of Drosophila ezoana in response to Nanda–Hamner light regimes. Percentage of diapausing flies plotted as function of period of the light/dark (LD) cycle. Each LD cycle consists of a fixed light phase of 10 h and a dark phase to make the required periodicity. Three distinct line‐types and symbols represent three separate experiments (runs R1–R3).

All three trials of the Nanda–Hamner experiment with *D. ezoana* showed a similar pattern of a gradual reduction in diapause incidence with an increasing period (T) of LD cycle (Fig. 3). Diapause incidence remained close to its maximum (approximately 90–100%) between a T of 18 and 36 h. From a T of 42 h and longer, a clear almost linear reduction in diapause incidence occurred. The Nanda–Hamner response in *D. ezoana* is therefore neither typically positive, nor typically negative.

### The critical night length for diapause induction in D. ezoana is independent of T: the flies appear to measure night length and to use a strongly damped oscillator to do this

To judge the putative involvement of a damped oscillator in the photoperiodic time measurement of *D. ezoana*, some newly designed T‐cycle experiments were applied. Night length was manipulated in two different ways: (i) as percentage of T and (ii) in absolute hours.

#### Experiment 1

**Figure 4:**
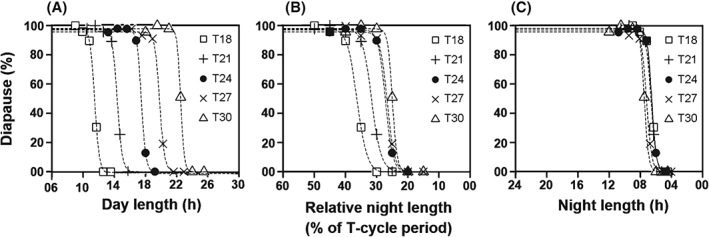
Diapause incidence of Drosophila ezoana in response to different photoperiods under T18, T21, T24, T27 and T30 (Experiment 1). The photoperiod was varied from 50% to 85% of T‐cycle period (Table 1) and diapause incidence was estimated for each photoperiod in each of the T‐cycles. (A) Percentage of diapausing flies plotted as function of absolute day length. (B) Percentage of diapausing flies plotted as function of relative night length (as percentage of T‐cycle period). (C) Percentage of diapausing flies plotted as function of absolute night length. In each plot, data points for the different T‐cycles are represented by different symbols. Dashed lines depict the nonlinear regression model fitted to the data.

In the first experiment, five different Ts were applied (T18, T21, T24, T27 and T30) and night length was varied as percentage of T (Table 1). The measured PPRCs showed the expected shape with a sharp reduction of diapause incidence when night length decreased below a certain percentage of T‐cycle period (Fig. 4). Most importantly, transition from diapause to reproduction appeared to differ among T‐cycles: the absolute day length (Fig. 4A) and the relative night length (Fig. 4B) corresponding to the transition (i.e. critical night length) decreased with T, as predicted from the external coincidence model under the assumption that ϕ_i_ is located near dawn under T < 24 h (Fig. 2). However, this trend does not readily imply the validity of the model because a qualitatively similar trend is also possible if the flies' measure a fixed night length. For example, a fly strain that needs a minimal night length of 6 h to enter diapause will have the critical night length at 33.3% of T in T18 and at 20% of T in T30. Therefore, the same diapause incidence data was plotted as a function of absolute night length (h) (Table 1). When doing so, the PPRCs under all five Ts almost completely coincided with each other (Fig. 4C). This suggests that the transition from diapause to reproduction occurs at the same night length of approximately 6–7 h irrespective of the T‐cycle period. These results thus indicate that *D. ezoana* flies measure night length for inducing diapause and use an hour‐glass clock or a strongly damped oscillator for photoperiodic time measurement, even though their Nanda–Hamner response was not typically ‘negative’.

#### Experiment 2

**Figure 5:**
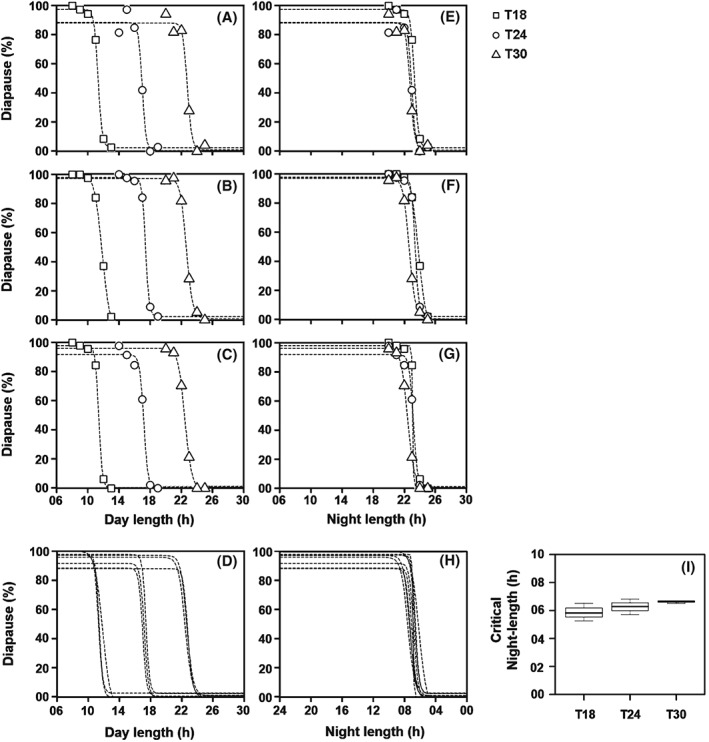
Diapause incidence in Drosophila ezoana in response to different day and night lengths under T18, T24 and T30 (Experiment 2). Night length was varied from 5 to 10 h in each of the T‐cycles (Table 2). Percentage of diapausing flies is plotted as function of absolute day length (A–C) and of absolute night length (E–G) in three separate experiments. Labelling as in Fig. 4. (D and H) Regression lines from all the three experiments plotted together demonstrate the differences in the transition from diapause to reproduction across T‐cycles with respect to different day lengths but an almost identical critical night length (CNL) at all Zeitgeber periods (T). (I) CNL as a function of T. The CNL was calculated as 50% in diapause from the regression lines shown in (H) (R language package ‘dose–response curve’; Ritz & Streibig, 2005; R Development Core Team, 2008).

A second experiment was performed to ensure that the flies indeed measure absolute night length, irrespective of T‐cycle period. Absolute night length was varied between 5 and 10 h under three different T‐cycle periods: T18, T24 and T30 (Table 2). This experiment was repeated three times (Fig. 5). The PPRCs of all three repetitions appeared to be very similar, with the plots of diapause incidence being different when plotted against day length (Fig. 5A–D) but completely coinciding when plotted against night length (Fig. 5E–H). The calculated mean critical night lengths under T18, T24 and T30 were 06.69, 07.02 and 07.03 h, respectively (Fig. 5I). The small trend of an increasing critical night length with increasing T turned out not to be significant (two‐way anova with ‘experiment’ as random factor: *F*_2,4_ = 3.38, *P* = 0.13). This experiment confirms that *D. ezoana* flies measure night length with an hour‐glass clock or a strongly damped oscillator. There is no evidence for the involvement of a sustained or weakly dampened circadian clock in the photoperiodic time measurement of this fly species.

### Female D. ezoana flies are only weakly rhythmic in DD

Circadian activity rhythms are commonly used as easily measureable ‘hands’ (outputs) of the circadian clock in the brain. Male and female fruit flies of the species *D. melanogaster* exhibit robust circadian activity rhythms when transferred from LD or from LL to DD conditions (Helfrich‐Förster, 2000, 2001). To determine whether this is also true for *D. ezoana* flies, 64 female flies were recorded for 14 days in DD after they had been transferred from LL.

**Figure 6:**
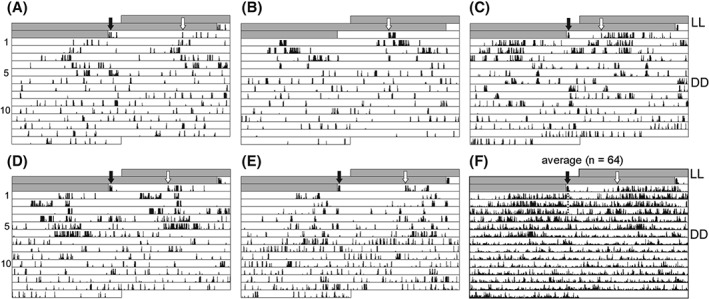
Representative individual actograms (A–E) and an average actogram (F) of Drosophila ezoana in constant darkness (DD). The average actogram (D) is calculated out of all 64 recorded flies. The flies were transferred from constant light (LL) to DD as indicated by the black arrow. The first onset of activity is indicated by a white arrow. In the average actogram, a grey dotted line indicates the end of activity during the few first days in DD.

It was found that *D. ezoana* flies were barely rhythmic under DD conditions (Fig. 6). On average, the flies showed weak rhythms for the first 2.5 ± 0.2 days (mean ± SD) after transfer to DD and subsequently became arrhythmic; only very few animals (Fig. 6) maintained some rhythmicity for up to 5 days. The initial rhythmicity of the flies can be seen in the average actogram calculated out of all 64 recorded flies (Fig. 6). The average actogram also shows that, on average, the end of the activity phase on the first day in DD coincides with the previous lights off. The same was observed in *D. melanogaster* flies after transfer from LL to DD (Helfrich‐Förster, 2001). Chi‐squared periodogram analysis of individual flies over the entire period in DD never revealed robust circadian periodicity in the data but, instead, highlighted multiple narrow spikes barely crossing the significance level. Such narrow spikes have also previously been observed in chi‐squared periodograms of *D. melanogaster* clock mutants and judged as false positives (Helfrich‐Förster, 1998; Klarsfeld *et al*., 2003). It is concluded that *D. ezoana* flies possess a strongly damped circadian clock that controls their activity rhythms.

## Discussion

The Nanda–Hamner experiments yield neither a typically positive, nor a typically negative response in *D. ezoana* (Fig. 3). Therefore, newly developed T‐cycle experiments are used to test the involvement of a sustained or damped circadian clock in photoperiodic time measurement. The T‐cycle study shows that the photoperiodic clock of the northern fruit fly species *D. ezoana* measures an absolute night length of approximately 7 h to induce diapause, irrespective of the photoperiod and the period of T‐cycles (Fig. 5). The absolute night‐length measurement strongly suggests that the time measurement in *D. ezoana* is achieved by a simple hour‐glass clock. However, such apparently hour‐glass based night‐length measurement may also be achieved by a weak and highly Zeitgeber responsive oscillator based clock through an ‘external coincidence’ principle as proposed by Pittendrigh (1966). The circadian oscillators of such clocks are considered to be completely (or mostly) damped by the end of the naturally occurring photoperiods and to be reset to the same phase at the onset of the night, causing ϕ_i_ to occur fixed hours afterwards. The night length is thus measured by sensing whether or not the ϕ_i_ is exposed to light. The absolute night length measurement in T‐cycle experiments thus suggests that a weak circadian oscillator may underlie the photoperiodic clock in *D. ezoana*. The present T‐cycle experiments appear to decipher the type of clock involved in photoperiodic time measurement, even in cases in which the classical Nanda–Hamner or Bünsow experiments lead to ambiguous results. Interestingly, *D. ezoana* flies appear to also utilize a strongly damped circadian clock for controlling locomotor activity rhythms. This suggests that the same weak circadian clock may be involved in photoperiodic time measurement and behavioural rhythm control. These observations in *D. ezoana* lead to three questions: (i) is the clock that measures night length identical to the clock that controls activity rhythms in all species showing diapause; (ii) is a weak, highly damped circadian clock better suited for photoperiodic time measurement than a sustained robust circadian clock; and (iii) what are the limitations of the newly developed T‐cycle experiments?

### Is the clock that measures night length identical to the clock that controls activity rhythms in all species showing diapause?

Circadian activity rhythms are a useful phase marker of the photoperiodic oscillator in many diapausing insects and their activity rhythms are therefore regarded as ‘hands of the photoperiodic clock’ (Kenny & Saunders, 1991), suggesting that the same clock controls activity rhythms and measures night length.

The results of the present study meet this expectation: *D. ezoana* flies appear to use a strongly damped oscillator for measuring night length and for controlling circadian activity rhythms. Nevertheless, there are superficial differences between the two clocks: the photoperiodic clock appears to damp within one cycle in DD, whereas the activity rhythm of *D. ezoana* flies free‐runs for a few cycles in DD. The reason for this difference may be a result of the different methods used in the diapause and activity rhythm assessment. The diapause assays measure the response of a population of flies, whereas locomotor activity recordings monitor the behaviour of single flies. Oscillator dampening is slightly different in individual flies. Some flies remain rhythmic for at least 5 days in DD, although the majority become arrhythmic after 2 days. As shown in Fig. 6(F), the average activity rhythm of the entire fly population, although still visible, is less clear than the rhythms of individual flies. Thus, the rhythmicity of a few flies disappears to some degree in the population. This may also explain the neither positive, nor negative Nanda–Hamner results. The clocks of the flies that remain rhythmic for a few days will respond approximately every 24 h to the light with diapause induction. However, the majority of the flies will barely respond. Because the measured diapause response is a mixture of the responses of the individual flies, results may be obtained that are neither positive, nor negative. The T‐cycle experiments are very different because they assess diapause induction not under free‐running conditions, as do the Nanda–Hamner experiments but under entrained conditions. As noted above, a highly damped oscillator or an hour‐glass clock will be reset every day at the onset of the night. Even weak circadian oscillators are highly responsive to LD cycles (Vitaterna *et al*., 2006; van der Leest *et al*., 2009). This may explain why even weak circadian clocks are reset by the light every day and thus measure night length. In summary, the present results are consistent with the hypothesis that the same clock governs diapause and rhythmic activity in *D. ezoana*.

**Figure 7:**
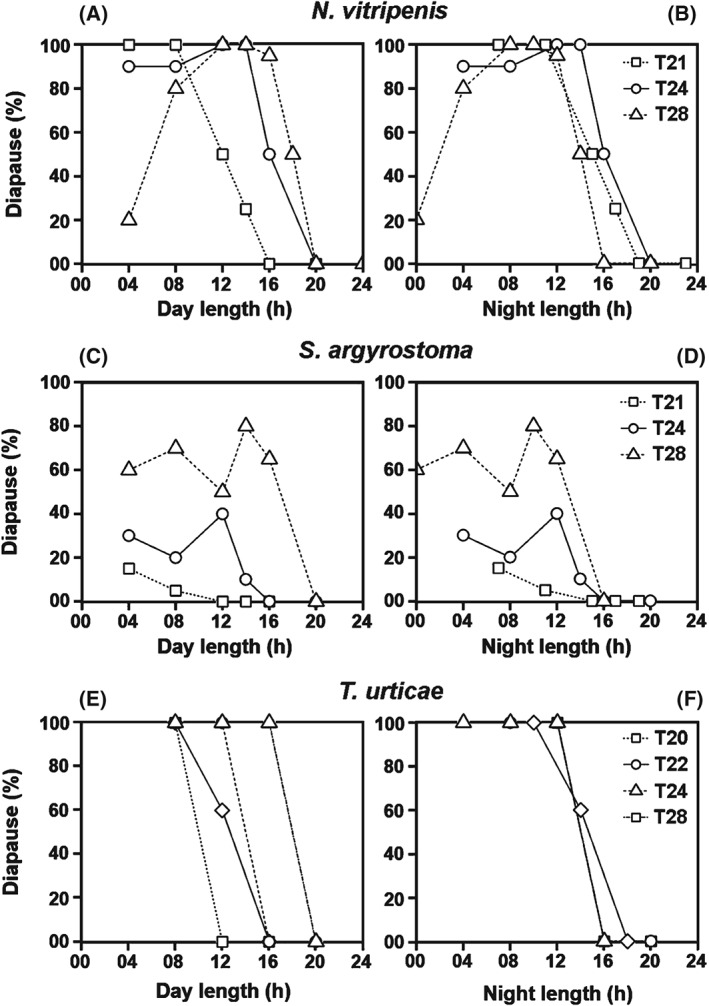
Diapause incidence in response to different day and night lengths under different T‐cycles in Nasonia vitripenis, Sarcophaga argyrostoma and Tetranychus urticae. The data reported were extracted from old Nanda–Hamner studies in the parasitic wasp N. vitripenis (Saunders, 1974), the flesh fly S. argyrostoma (Saunders, 1973) and the spider mite T. urticae (Vaz Nunes & Veerman, 1986). These studies conducted Nanda–Hamner experiments using a range of photoperiod conditions, thus allowing us to extract diapause incidence for more than one combination of photoperiods and night lengths for each LD periodicity. The extracted diapause incidence data were then plotted as a function of day length (A, C, E) and night length (B, D, F). Distinct line‐types and symbols in each plot represent different T‐cycle periods.

Does this also hold true for species that exhibit ‘positive’ Nanda–Hamner responses and are thus expected to possess strong self‐sustained oscillator‐based photoperiodic clocks (Saunders & Lewis, 1987)? Diapausing insect species, such as the parasitic wasp *Nasonia vitripenis*, the flesh fly *Sarcophaga argyrostoma* and the spider mite *Tetranychus urticae*, are known to exhibit robust positive Nanda–Hamner responses (Saunders, 1973, 1974; Vaz Nunes & Veerman, 1986). *Nasonia vitripennis* additionally displays robust circadian activity rhythms (Bertossa *et al*., 2013), highlighting the assumption that the same robust circadian oscillator may control activity and measure photoperiod. If true, these insects should also show a prominent change in critical night length under the newly designed T‐cycle experiments described in the present study. To test this, the diapause incidence data are extracted from the published studies involving these species (Saunders, 1973, 1974; Vaz Nunes & Veerman, 1986) and re‐plotted as a function of day length (Fig. 7A, C, E) and night length (Figure 7B, D, F) for each T‐cycle separately, as carried out for *D. ezoana* in Fig. 4. In all three species, the diapause to nondiapause transition occurs at different day lengths under different T‐cycles (Figure 7A, C, E) but only in *S. argyrostoma* does it also occur at different night lengths (Fig. 7D). In *N. vitripennis*, the transition shows a tendency to occur at the same night length, irrespective of the T‐cycle period (Fig. 7B) and, in *T. urticae*, the curves at the four T‐cycles largely overlap (Fig. 7F). This suggests that at least *T. urticae* and perhaps also *N. vitripennis* may use a strongly damped circadian oscillator to measure night length despite the positive Nanda–Hamner responses. If true, this also implies that this oscillator does not coincide with the one controlling activity rhythms. It is nevertheless too early to draw any definitive conclusions because the extraction of the diapause incidence data from the published studies may still be imprecise. New studies with the three species in the newly designed T‐cycle experiments may help to solve the issue.

At present, it is not clear whether a positive Nanda–Hamner response is a proof for the involvement of a sustained circadian oscillator in photoperiodic time measurement or whether it is exclusively the result of a noncausal circadian influence on the diapause physiology, as suggested by Pittendrigh & Minis (1964) and Pittendrigh (1972). The T‐cycle experiments will help to answer this important question. Combined with activity rhythm recordings, these experiments will also help to clarify whether the clock controlling the behavioural rhythms is identical to the clock measuring night length for diapause induction.

There is a remarkable debate in the literature about this issue. Saunders *et al*. (1989) show that *D. melanogaster* flies remain capable of measuring day length in the absence of the canonical circadian clock gene *period* that is essential for normal activity rhythms, which suggests that the circadian clock is not involved in measuring day length. However, more recent studies indicate that the circadian clock gene *timeless* determines general diapause incidence (Tauber *et al*., 2007). The *timeless* gene is also shown to be involved in the photoperiodic response of the fly *Chymomyza costata* (Pavelka *et al*., 2003) and the *period* gene in the cricket *Modicogryllus siamensis* (Sakamoto *et al*., 2009). Furthermore, the clock genes *period*, *cycle*, mammalian type *cryptochrome* and *clock* are important for diapause in the bean bug *Riptortus pedestris* (Ikeno *et al*., 2010, 2011, 2013). Perhaps the most compelling evidence that not only the circadian clock genes, but also the circadian clock neurones driving activity rhythms are essential for photoperiodism comes from ablation experiments in the blow fly *Protophormia terraenovae* (Shiga & Numata, 2009). *Protophormia terraenovae* shows robust circadian rhythms and even the anatomical connections from certain circadian clock neurones to the diapause inducing neurosecretory cells in the brain are reported (Shiga & Numata, 2000; Hamanaka *et al*., 2005). So far, there are more studies in favour of the involvement of the same robust oscillator in circadian activity control and night length measurement than there are against it. The pitcher‐plant mosquito *Wyeomyia smithii* is perhaps the best example arguing against a role of the same oscillator in both processes. During the summer, the larvae of *W. smithii* live as top‐level predators in the water that is contained by the purple pitcher plant *Sarracenia purpurea*. In response to short‐day conditions, the third‐ or fourth‐instar *W. smithii* larvae enter diapause (Bradshaw & Lounibos, 1972). Formal, classical experiments show that a weak dampening oscillator participates in photoperiodic induction of diapause in *W. smithii* (Wegis *et al*., 1997; Bradshaw *et al*., 1998) and also that the influence of this oscillator decreases in populations living at high latitudes (Bradshaw & Holzapfel, 2001). Bradshaw & Holzapfel (2001) assume that the circadian clock of *W. smithii* is robust and argue that it would appear to be maladaptive to couple the robust circadian clock to the highly variable and rapidly evolving photoperiodic calendar. Instead, they propose that photoperiodic timing is a process separate from the circadian clock and capable of independent evolution without disrupting the temporal organization of daily events (Emerson *et al*., 2009). This hypothesis receives support from findings obtained in *Drosophila littoralis* flies, which show no apparent relationship between critical photoperiod and circadian gating of adult emergence over a latitudinal cline (Lankinen, 1986) and in which laboratory selection results in independent counter‐current ‘evolutions’ of critical photoperiod and circadian controlled adult emergence (Lankinen & Forsman, 2006). However, does the circadian clock necessarily determine the critical photoperiod or the *critical night length*? Perhaps the circadian clock only provides the necessary time reference to determine day or night length and it is environmental factors such as temperature that set the final critical photoperiod. Furthermore, is it true that all species with robust photoperiodic diapause also have robust circadian clocks? The circadian activity of *W. smithii* and *T. urticae* has not yet been investigated and several northern fly species with strong photoperiodism, such as *D. ezoana* (present study), *D. littoralis* (Riihimaa, 1996; Lankinen & Forsman, 2006) and *Drosophila montana* (Kauranen *et al*., 2012, 2016), have extremely weak circadian rhythms. Thus, the assumption that all species with robust diapause have robust circadian clocks may still be wrong, leaving the possibility open that the same weak circadian clock is involved in both processes.

### Is a weak, highly damped circadian clock better suited for photoperiodic time measurement than a sustained robust circadian clock?

As noted above, several fly species living at high latitudes show weak circadian rhythms. Such observations are also reported for non‐insect species. Reindeers recorded under long arctic summer days lack robust activity rhythms and other physiological circadian rhythms that are clearly oscillating in hamsters and other mammal with robust clocks (Van Oort *et al*., 2005; Lu *et al*., 2010), suggesting that weak circadian clocks are common in animals living in the north. Possibly, weak circadian clocks, or even hour‐glass clocks are of advantage in the north. Weak circadian clocks are more plastic (flexible), can more easily synchronize to different LD cycles, and phase‐shift to light‐pulses more readily compared with strong circadian clocks (Vitaterna *et al*., 2006; van der Leest *et al*., 2009; Abraham *et al*., 2010). In the north, the photoperiod lengthens dramatically from spring to summer and radically shortens again in autumn. A weak circadian clock or an hour‐glass clock is assumed to easily synchronize to these changing photoperiods, whereas a strong circadian clock might have difficulties. At the same time, weak circadian or hour‐glass clocks are satisfactorily ticking under LD cycles and can serve as time reference for photoperiodism. Thus, they appear better suited to measure night length in the changing environment in the north.

In different northern *Drosophila* species with weak circadian rhythms, the clock in the brain controlling these rhythms exhibits clear morphological differences compared with the brain clock of the robustly rhythmic *D. melanogaster* flies (Hermann *et al*., 2013; Kauranen *et al*., 2013). The brain clocks of *Drosophila virilis*, *D. ezoana*, *D. montana* and *D. littoralis* lack the neuropeptide pigment‐dispersing factor, PDF, in one set of lateral clock neurones. PDF is known to be essential for robust circadian activity rhythms of *D. melanogaster* in DD (Helfrich‐Förster, 1998; Renn *et al*., 1999). Furthermore, the PDF‐expressing clock neurones control the morning activity of the flies, whereas other clock neurones control evening activity. Thus, *D. melanogaster* flies (and also *S. argyrostoma* and *P. terraenovae* flies) appear to possess two oscillators, whereas the aforementioned northern *Drosophila* species retain only one oscillator that appears not to be sufficiently strong to provoke robust circadian rhythms. This one weak oscillator (hour‐glass) may, however, be perfectly suited for measuring night length via the external coincidence model.

### Limitations of the T‐cycle experiments

There is also another way of measuring time known as the internal coincidence model (Saunders, 1974, 1978). The internal coincidence model relies on the presence of two circadian oscillators, namely the M and E oscillators (Pittendrigh & Daan, 1976). The M and E oscillators track dawn and dusk, respectively, in several insect species. Consequently, they have different phase relationships to each other under short winter days and long summer days. Under short winter days, their phases are close together and, under long summer days, their phases are far apart. These internal phase differences can be used perfectly to determine night length or day length. Thus, measuring night length with the help of two robust oscillators is another way of reliably inducing diapause when the days become shorter. Actually, *N. vitripennis*, which shows positive Nanda–Hamner responses, appears to use the internal coincidence model for measuring night length (Saunders, 1974). The newly developed T‐cycle experiments that are described in the present study will also change the phases of the two oscillators: the phases of both will occur later under short Ts and earlier under long Ts. However, as long as the phases of both oscillators are similarly shifted, the mutual phase relationship between M and E oscillator remains the same and, consequently, the critical night will not be influenced by T. This means that the T‐cycle experiments cannot reliably distinguish between robust oscillators in the internal coincidence model and damped oscillators in the external coincidence model.

Nevertheless, together with recordings of the activity rhythms, it should be possible to distinguish between these two possibilities. Therefore, it is considered that the proposed T‐cycle experiments will be very useful for clarifying whether damped or robust self‐sustained oscillators measure night length and control diapause. Furthermore, the T‐cycle experiments are suited to resolving questions about separate or identical clocks controlling diapause and circadian activity rhythms.
